# Rationale for the clinical use of adipose-derived mesenchymal stem cells for COVID-19 patients

**DOI:** 10.1186/s12967-020-02380-2

**Published:** 2020-05-18

**Authors:** Christopher J. Rogers, Robert J. Harman, Bruce A. Bunnell, Martin A. Schreiber, Charlie Xiang, Fu-Sheng Wang, Antonio F. Santidrian, Boris R. Minev

**Affiliations:** 1 Personalized Stem Cells, Inc, Poway, CA USA; 2 Center for Stem Cell Research and Regenerative Medicine, Tulane University School of Medicine, New Orleans, LA USA; 3 Department of Surgery, Oregon Health and Science University, Portland, OR USA; 4 State Key Laboratory for Diagnosis and Treatment of Infectious Diseases, National Clinical Research Center for Infectious Diseases, The First Affiliated Hospital, College of Medicine, Zhejiang University, Hangzhou, 310003 China; 5 Treatment and Research Center for Infectious Diseases, The Fifth Medical Center, Beijing, 100039 China; 6 Calidi Biotherapeutics, Inc., San Diego, CA USA; 7 Department of Radiation Medicine and Applied Sciences, Moores UCSD Cancer Center, San Diego, CA USA

**Keywords:** COVID-19, SARS-CoV-2, ARDS, Pneumonia, Mesenchymal stem cells

## Abstract

In late 2019, a novel coronavirus (SARS-CoV-2) emerged in Wuhan, capital city of Hubei province in China. Cases of SARS-CoV-2 infection quickly grew by several thousand per day. Less than 100 days later, the World Health Organization declared that the rapidly spreading viral outbreak had become a global pandemic. Coronavirus disease 2019 (COVID-19) is typically associated with fever and respiratory symptoms. It often progresses to severe respiratory distress and multi-organ failure which carry a high mortality rate. Older patients or those with medical comorbidities are at greater risk for severe disease. Inflammation, pulmonary edema and an over-reactive immune response can lead to hypoxia, respiratory distress and lung damage. Mesenchymal stromal/stem cells (MSCs) possess potent and broad-ranging immunomodulatory activities. Multiple in vivo studies in animal models and ex vivo human lung models have demonstrated the MSC’s impressive capacity to inhibit lung damage, reduce inflammation, dampen immune responses and aid with alveolar fluid clearance. Additionally, MSCs produce molecules that are antimicrobial and reduce pain. Upon administration by the intravenous route, the cells travel directly to the lungs where the majority are sequestered, a great benefit for the treatment of pulmonary disease. The in vivo safety of local and intravenous administration of MSCs has been demonstrated in multiple human clinical trials, including studies of acute respiratory distress syndrome (ARDS). Recently, the application of MSCs in the context of ongoing COVID-19 disease and other viral respiratory illnesses has demonstrated reduced patient mortality and, in some cases, improved long-term pulmonary function. Adipose-derived stem cells (ASC), an abundant type of MSC, are proposed as a therapeutic option for the treatment of COVID-19 in order to reduce morbidity and mortality. Additionally, when proven to be safe and effective, ASC treatments may reduce the demand on critical hospital resources. The ongoing COVID-19 outbreak has resulted in significant healthcare and socioeconomic burdens across the globe. There is a desperate need for safe and effective treatments. Cellular based therapies hold great promise for the treatment of COVID-19. This literature summary reviews the scientific rationale and need for clinical studies of adipose-derived stem cells and other types of mesenchymal stem cells in the treatment of patients who suffer with COVID-19.

## Background

In late December 2019, multiple cases of severe respiratory distress with an unknown cause were reported in Wuhan, China. In January, a highly contagious novel coronavirus (now called SARS-CoV-2) was identified as the cause which rapidly spread resulting in a global pandemic. As of April 20, 2020, the World Health Organization has reported over 2,400,000 confirmed cases and more than 165,000 deaths in more than 180 countries and 200 territories. At the present time there is an urgent need to develop safe and effective treatments for COVID-19 patients because, currently, no such therapy exists.

The healthcare industry is using every viable option to suppress the global threat, including vitamins, pharmacologics, convalescent plasma and vaccine development. While numerous therapeutic and preventative avenues are being investigated, mesenchymal stem cells (MSCs) offer a compelling treatment option. In recent months, there has been increased interest in the clinical trial sector for the use of MSC therapies in COVID-19 patients. MSCs have received particular attention because of their ability to inhibit inflammation and cytokine storms as demonstrated in several in vitro and in vivo models [1, 2]. Early-stage studies of MSC treatment in acute respiratory distress syndrome (ARDS) models have reported improvements in the lung microenvironment, inhibition of the over-active immune system, promotion of tissue repair, protection of lung alveoli epithelial cells, prevention of pulmonary fibrosis and the preservation of long-term pulmonary function. MSCs also secrete molecules that are antibacterial [3], anti-viral [4] and analgesic [5].

Adipose-derived stem cells (ASC) are an abundant type of MSC that expresses these important characteristics. ASCs are proposed as a relatively safe therapeutic tool to treat COVID-19 patients with the goals of reduction in mortality and morbidity. Treatment with ASCs may also reduce the demand on critical hospital resources such as intensive care unit (ICU) beds and mechanical ventilators.

## Pathogenesis of SARS-CoV-2 infection (COVID-19)

Common clinical features of COVID-19 include fever, headache, malaise, cough, bone pain, myalgias, anosmia, impaired taste and respiratory distress. Similar to SARS in 2003, this infectious disease results in a high probability of ICU admission and mortality [6–8]. The pathogenesis of SARS-CoV-2 infection includes the recognition of the angiotensin-converting enzyme 2 receptor (ACE2) by the virus spike protein and priming of the spike protein by the cellular transmembrane protease, serine 2 (TMPRSS2) facilitating host cell entry and spread [9–11]. Severe respiratory illness is a primary outcome of SARS-CoV-2 infection because the ACE2 receptor is widely expressed on alveolar type II cells and capillary endothelial cells. Also, the alveolar cells are known to express TMPRSS2 [12]. Viral lung infection results in the generation of a cytokine storm, as defined by the rapid efflux of a large number of cytokines. The elevated levels of pro-inflammatory cytokines drive extensive lung tissue edema, air exchange dysfunction, acute respiratory distress, secondary infection and death in 0.7 to 17.3% of patients [13].

Due to the rapid progression of the cytokine cascade, critically ill COVID-19 patients develop acute respiratory distress syndrome (ARDS) and may require respiratory support. Yang et al. reported that 67% of critically ill patients develop ARDS and the mortality of these patients is considerable. Survival time after ICU admission is generally 1–2 weeks. ARDS patients older than 65 years with comorbidities associated with immune dysregulation, such as diabetes or obesity have a higher mortality rate. Xu et al. confirmed that COVID-19 patients with severe pneumonia died from severe infection with ARDS in biopsy samples at autopsy [14]. ACE2 receptors are also expressed in the heart, liver, kidney and digestive organs. Such patients are not only afflicted with ARDS, but other complications as well such as myocardial damage, arrhythmia, acute kidney injury and multiple organ dysfunction syndrome.

## Acute respiratory distress syndrome (ARDS)

ARDS is a multi-factorial syndrome of severe lung injury characterized by hypoxemia, pulmonary edema, diffuse alveolar damage and multiple organ failure [15–17]. ARDS from all causes affects approximately 200,000 Americans annually and carries a mortality rate of 30–50% [18]. Of those patients who survive, significant morbidity occurs due to neuromuscular weakness, neuropathy, myopathy, residual lung fibrosis and cognitive issues. These may persist even five years after recovery from ARDS, resulting in increased healthcare utilization and costs [19].

The current definition of ARDS is clinical, based on chest X-ray finding of bilateral infiltrates, the timing of initial injury, absence of cardiogenic pulmonary edema and measurement of hypoxemia. The underlying causes of ARDS vary, but patients follow a similar clinical pattern of lung injury [18]. ARDS is classified utilizing the Berlin Definition which is based on the degree of hypoxemia as gauged by PaO2/FiO2 ratio. Mild ARDS, previously defined as acute lung injury (ALI), is defined by a PaO2/FiO2 (PF) ratio of 200-300. Moderate ARDS is defined by PF ratio of 100-200 and patients with severe ARDS have a PF ratio < 100. The mortality rate for mild, moderate and severe ARDS is 27%, 32% and 45%, respectively [20].

ARDS-associated lung injury is caused by intense pulmonary and systemic inflammation with neutrophil and macrophage invasion into the alveolar spaces. The localized release of pro-inflammatory cytokines such as IL-6, IL-1β, IL-8, and TNF-α leads to damage of the endothelial and epithelial lung tissues. The inflammatory environment within the lung enhances the production of reactive oxygen species, impairs lung barrier function, increases vascular permeability, promotes the accumulation of alveolar proteinaceous exudate and eventually results in pulmonary fibrosis [21, 22].

The neutrophils may migrate from inflamed tissues to other organs causing widespread organ dysfunction. Neutrophils kill microorganisms by phagocytosis or the release of neutrophil extracellular traps (NETs), which are comprised of a core of chromatin DNA and histones surrounded by proteases, reactive oxygen species-generating enzymes and anti-microbial proteins. The excessive NETs release directly damages lung tissues [23].

Current efforts for the management and treatment of this deadly disease remain supportive with efforts to reduce ventilator-induced lung injury and permit an endogenous repair process to help with recovery from lung injury. There is some evidence that neuromuscular blockade and prone positioning improves survival in ARDS patients, but the evidence is not definitive [24, 25]. Management with mechanical ventilation and fluid-restriction provides organ support while minimizing iatrogenic harm [22]. Several pharmacological approaches have been tried including glucocorticoids, surfactants, inhaled nitric oxide, antioxidants and protease inhibitors. Unfortunately, these treatments have been found to be completely ineffective [26, 27]. Currently, no direct therapies for ARDS exist.

## Mesenchymal stromal/stem cells (MSCs)

In 1968, Friedenstein et al. first isolated mesenchymal stem cells from bone marrow (BM-MSCs), which was then termed a colony-forming unit fibroblast (CFU-F) [28]. These plastic-adherent bone marrow stromal cells were denoted as CFU-F due to their fibroblastic appearance and ability to form colonies in vitro. In 1991, Caplan termed these cells mesenchymal stem cells (MSCs) due to their mesenchyme origin during embryonic development noting an ability to differentiate into osteoblasts, adipocytes, and chondrocytes [29]. In response to the varying nomenclature used to characterize MSCs, in 2005 the International Society of Cellular Therapy (ISCT) proposed that these stromal cells be referred to as multipotent mesenchymal stromal cells and the term be reserved for cells that meet the following criteria [30, 31]:

1. Plastic adherence in standard culture conditions.
2. Surface marker expression of CD105, CD73 and CD90.
3. Lack expression of CD45, CD34, CD14 or CD11b, CD79 or CD19 and HLA-DR.
4. Differentiation potential toward osteoblasts, adipocytes, and chondrocytes in vitro.

In 2017, Caplan suggested that the name “mesenchymal stem cells” be changed to “medicinal signaling cells” to more accurately reflect the fact that, in vivo, these cells secrete bioactive factors that are immunomodulatory and trophic, meaning that these cells make medicinal drugs in situ [32]. Below we describe the mechanisms of action of mesenchymal stem cells (MSCs).

## How do MSCs function?

MSCs are known to function via several mechanisms relevant to acute lung injury.

- When administered intravenously:
  - MSCs sequester in the lung.
  - MSCs are immune-evasive.
  - MSCs are immune-modulatory.
- Specific mechanisms of therapeutic action:
  - Anti-inflammatory.
  - Antibacterial.
  - Antiviral.
  - Lung fibrosis inhibition.
  - Lung tissue regeneration.
  - Anti-apoptotic of injured cells.
  - Alveolar fluid clearance.
  - Extracellular vesicle production.

### MSCs are abundant in adipose tissue and highly potent

Human ASCs have significant advantages over MSCs derived from other sources because they are obtained from a minimally invasive lipoaspiration procedure [33]. The MSC concentration in adipose is greater than all other tissues in the body [34, 35] and the potency is maintained with age of the donor [36], unlike bone marrow derived MSCs [37, 38]. Significant numbers of ASCs can be obtained due to accessibility to the subcutaneous adipose tissue and the volume that can easily be extracted. Taken together, the ASC has advantages in both autologous use and allogeneic use. Significant anti-inflammatory effects have been confirmed in many veterinary and human clinical studies [39–41].

### Infused MSCs are sequestered in the lung

Intravenous infusion of MSCs are known to accumulate in the lungs, a great benefit for treatment of pulmonary disease, where they secrete numerous paracrine factors that can play a significant role in protecting or rejuvenating alveolar epithelial cells, counteract fibrosis and improve lung function [42]. Following intravenous administration, only a small fraction of MSCs engraft in target organs, as they become entrapped in the lung’s microvasculature [43–45]. Intravenous administration of MSCs may be useful for patients with multi-organ disease due to the MSC’s ability to home to and act on other injured organs such as the heart, liver and kidney [46].

### MSCs are immune-evasive

Culture-expanded MSCs express low levels of MHC class I, and no MHC class II or co-stimulatory molecules B7-1, B7-2 or CD40 [47]. ASCs express even lower levels of HLA class-I molecule when compared with BM-MSCs [48].

### Modulation of the immune system

It is well established that the primary mechanism by which MSCs exert their therapeutic effects is via secretion of soluble factors known as the secretome. The MSC secretome is comprised of an array of bioactive molecules that includes cytokines, chemokines, growth factors, angiogenic factors and extracellular vesicles. MSCs may interact directly with immune cells and execute paracrine modulation of the immune response by the release of cytokines such as IL-10, IL-1RA, TGF-β, and the production of indoleamine 2,3 dioxygenase (IDO) and nitric oxide [49].

These mechanisms modulate the proliferation and activation of naïve and effector T cells, natural killer (NK) cells and mononuclear cells towards an anti-inflammatory phenotype. Modulation of T cell function includes the inhibition of the Th17 response, the induction of regulatory T cells, and the shift from a Th1 to the Th2 cell phenotype. This process is regulated by anti-inflammatory cytokines, including IL-10 and TGF-β, growth factors such as HGF, and additional soluble factors like PGE2 and the inhibition of cytokines like IL-4 and IL-13 [50, 51].

Improved outcomes after MSC infusions in multiple medical conditions have been primarily attributed to the MSC-based production of paracrine factors which interact with immune cells resulting eventually in immunomodulation [52–55]. Several studies have demonstrated that MSCs regulate the activity of both the innate and adaptive immune response via direct cell–cell contact and trophic factors. COVID-19 patients have been shown to possess reduced regulatory T cells counts, especially in severe cases [56]. MSCs, and in particular, ASCs increase the generation of regulatory T cells (Treg) [57–59]. ASCs have been shown to promote the development of Tregs more effectively than BM-MSCs or Wharton jelly-derived MSCs (WJ-MSC) [60]. MSCs also demonstrate inhibition of the proliferation, activation and maturation of CD19^+^ B cells, CD4^+^ Th1 cells, CD8^+^ T cells, macrophages, monocytes and neutrophils [61, 62]. The ASC’s immunomodulation of dendritic cell differentiation has been shown to be more effective than BM-MSCs [63]. MSCs, when engulfed by immune cells such as monocytic cells of predominantly non-classical Ly6C_low_ phenotype, induce phenotypical and functional changes in monocytes, which subsequently modulate cells of the adaptive immune system [64, 65].

Excessive neutrophil activation in sepsis causes injury to lung tissue and other organs. Improvement in organ damage associated with sepsis correlates with the reduction of neutrophil infiltration in target organs. In a murine model of sepsis, systemic infusion of allogeneic ASCs ameliorated clinical and histopathologic evidence of disease severity and was associated with IL-10-secreting Treg cells activation [66]. MSCs were also shown to inhibit neutrophil oxidative burst, decrease extracellular release of myeloperoxidase and elastase and strongly attenuate neutrophil mediated damage in an in vivo model of vasculitis [63, 67, 68].

### Production of anti-inflammatory molecules

The administration of MSCs has demonstrated anti-inflammatory effects in many acute lung injury models [69–72]. MSCs shift inflammation from an overwhelming release of pro-inflammatory cytokines including IL-1β, IL-6, MCP-1, MIP-2, CXCL-1, CXCL-2, TNF-β, IL-12, IL-17 or type II IFN-γ and proteases like MMP-2, MMP-9 and MMP-12 to an anti-inflammatory status. Released anti-inflammatory cytokines include IL-4, IL-10, TGF- α, CCL18, prostaglandin E2, IDO, nitric oxide and lipoxin A4. This shift promotes the resolution of inflammation and tissue repair [73, 74].

MSCs shift the phenotype and function of antigen-presenting cells, including dendritic cells, B lymphocytes and macrophages and prevent neutrophil extracellular trap formation [75]. The phenotype switch in macrophages leads to a return from the M1 state to the “anti-inflammatory” M2 state. This switch is induced by the inflammatory milieu of bacterial infection which fosters inflammation by the release of pro-inflammatory factors, including cytokines and proteolytic enzymes.

In the liver, MSCs regulate the NLRP3 inflammasome which regulates the activation of capsase-1 and the subsequent inflammatory response to infectious microbes and molecules in Kupffer cells via secretion of PGE2. This leads to increased IL-10 production by the Kupffer cells and alleviation of the inflammatory response and organ dysfunction [76].

### MSCs are anti-bacterial and promote macrophage removal of bacteria

MSCs possess innate antimicrobial properties, which have been demonstrated both in vitro and in small animal models. Direct bacterial clearance by MSCs is mediated by LL-37 [3] by beta-defensin-2 via the toll-like receptor 4 (TLR-4) signaling pathway [77] and lipocalin-2 [78]. MSCs have been shown to increase the phagocytic activity of macrophages when reprograming them from the pro-inflammatory to an anti-inflammatory phenotype [79, 80].

### MSCs are anti-viral

MSCs suppress viral replication, viral shedding and virus-induced lung epithelial cell (LEC) damage [4]. Khatri et al. [4] demonstrated that MSC-derived extracellular vesicles (MSC-EVs) promote both anti-inflammatory and anti-viral properties via transfer of RNAs from EVs to LECs. In a porcine model of influenza viral pneumonia, they showed that intratracheal administration of MSC-EVs significantly reduced virus entry and replication in LECs.

Virus shedding in nasal swabs and virus titers in lung lysate were reduced by 100-fold in the MSC-EV treated group. Influenza virus induced LEC apoptosis and red blood cell hemagglutination were significantly reduced by MSC-EVs as well.

Influenza virus replication is further inhibited by MSC production of indoleamine 2,3-dioxygenase (IDO) [81] and LL37 by viral membrane degradation [82]. IDO has also been shown to suppress viral replication in hepatitis B, herpes simplex virus (HSV), cytomegalovirus (CMV) and measles virus [83–86]. Unregulated inflammation in influenza virus infection leads to extensive lung damage. MSC-EVs are known to decrease production of proinflammatory cytokines and chemokines and increase production of anti-inflammatory IL-10 [4]. MSCs interact with immune cells and promote T-regulatory cells (Tregs) which improves influenza virus clearance [87, 88]. MSC-EV inhibition of viral replication has also been demonstrated in hepatitis C virus (HCV) infected fibroblasts [89].

### MSCs inhibit lung fibrosis and scar formation

Fibroblast and myofibroblast deposition are promoted during epithelial tissue repair. Increased cellular matrix protein synthesis leads to low tissue compliance, lung parenchymal scarring and long-term loss of function [90]. Neutrophil and macrophage recruitment in the lung activates profibrotic proteins which promote collagen release from fibroblasts. Lung tissue obtained from patients with fibrotic lung diseases contained an enhanced number of MSCs [91]. Animal models have demonstrated the positive effects of MSCs when applied early to ameliorate inflammation and moderate fibrotic lung tissue remodeling [92].

Keratinocyte growth factor (KGF) is involved in pulmonary epithelial repair, as shown in a study in which BM-MSCs modified to express KGF proved effective against bleomycin-induced pulmonary fibrosis in a murine model [93–95]. Induced pluripotent stem cell (iPSC) conditioned media, known to contain hepatocyte growth factor (HGF), attenuates fibrosis and promotes alveolar epithelium repair [96]. The administration of UC-MSCs has also been shown to reduce collagen concentrations in the lung and to inhibit the expression of transforming growth factor-beta (TGF-β), interferon-gamma (IFN-γ), macrophage migratory inhibitory factor and tumor necrosis factor-alpha (TNF-α) in a murine model of bleomycin-induced acute lung injury [97].

### MSCs secrete molecules that are mitogenic and anti-apoptotic

MSCs exert an anti-apoptotic effect due to the secretion of bioactive factors, such as vascular endothelial growth factor (VEGF), insulin growth factor (IGF), hepatocyte growth factor (HGF), neurotrophin-3 and nerve growth factor, as well as through mitochondrial and microvesicle transfer [98–101]. Lung injury is also ameliorated by autophagy which may result from the MSC response to oxidative stress, cytoprotection and phosphoinositide 3-kinase/protein kinase B (P13K/Akt) signaling pathway [102–105].

### MSCs clear alveolar fluid from the lungs

Alveolar type II (ATII) cells make up approximately 2–5% of the alveolar surface area and have several important functions. They produce surfactant and serve as progenitor cells for the alveolar epithelium. Alveolar fluid is driven by the movement of sodium and chloride ions through epithelial transporters down an osmotic gradient to exit the alveoli and maintain dry airspaces. Patients with ARDS have impaired alveolar fluid clearance (AFC) which is associated with higher morbidity and mortality [106]. Multiple studies confirm that MSC interaction with sodium and chloride ion channels enhances AFC and promotes the resolution of pulmonary edema [107]. Loy et al. showed that MSCs improved AFC and alveolar protein permeability (APP) in an Influenza A (H5N1) virus-associated acute lung injury. Interestingly, combined hepatocyte growth factor (HGF) and angiopoietin 1(Ang-1) restored AFC and APP, but the combination was less effective than the MSCs alone [108].

Lee et al. studied the therapeutic capacity of human BM-MSCs to restore alveolar epithelial fluid transport and lung fluid balance from acute lung injury in an ex vivo perfused human lung preparation injured by *E. coli* endotoxin. They showed reduced extravascular lung edema, improved lung endothelial barrier permeability and restoration of alveolar fluid clearance. The effect was mediated in part by the secretion of KGF which helped restore sodium dependent alveolar fluid transport [109]. Using ex vivo lung perfusion in human lungs that had been rejected for transplantation, Genai and colleagues demonstrated that microvesicles derived from human BM-MSCs also increased alveolar fluid clearance and improved airway and hemodynamic parameters compared to perfusion alone [110]. Alveolar fluid clearance is promoted by keratinocyte growth factor (KGF) and KGF repair can be facilitated by MSC derived microvesicles that transfer mRNA [111, 112].

Fang et al. performed a genome-wide exploratory analysis of human alveolar type II cell gene expression in response to stimulation with pro-inflammatory cytokines in the presence or absence of human MSCs. They reported that stimulation of ATII cells with pro-inflammatory cytokines increased expression of inflammatory genes and downregulated genes related to surfactant function and alveolar fluid clearance. In the presence of MSCs, ATII cells upregulated the genes coding surfactant protein and downregulated genes associated with apoptosis which has been linked to ARDS pathogenesis. The MSCs also induced ATII cells to upregulate genes involved in extracellular matrix modification and other genes related to injury repair [113].

Xiang et al. reported the therapeutic potential of human menstrual blood-derived MSCs to reduce lipopolysaccharide (LPS)-induced acute lung injury (ALI) inflammation in mice and promote damaged repair of lung functions [114]. They showed that MSCs not only improved pulmonary microvascular permeability, but also decreased histopathological damage mediated through the downregulation of IL-1β and up-regulation of IL-10 expression in bronchoalveolar lavage fluid (BALF). Additionally, MSCs improved the activity of BEAS-2B in human lung epithelial cells and inhibited LPS induced cell apoptosis.

Chan et al. compared the extent to which avian influenza A/H5N1 virus and seasonal influenza A/H1N1 virus impair alveolar fluid clearance and protein permeability in an in vitro murine model of acute lung injury [115]. The alveolar epithelium’s protein permeability and fluid clearance were dysregulated by soluble immune mediators released after infection with avian (A/Hong Kong/483/97, H5N1) but not seasonal (A/Hong Kong/54/98, H1N1) influenza virus. They demonstrated that these effects were prevented or reduced by the infusion of MSCs, which improved survival. Finally, the secretion of angiopoietin-1 by MSCs has been shown to reduce lung protein permeability which acts to stabilize endothelial cells [113, 116].

### MSCs produce extracellular vesicles

Recent studies show that MSCs produce extracellular vesicles (EVs) that can help ameliorate acute lung injury [117, 118]. EVs comprise exosomes, microvesicles (MVs) and apoptotic bodies. MVs form directly by budding from the cell membrane and are 100 nm to 1000 nm in size. They are abundant in selectins, integrins, CD-40, phosphatidylserine and metalloproteinases. Apoptotic bodies are fragments of dying cells which form and are released in the extracellular space by plasma membrane budding during the apoptotic process. They are irregular in shape and are between 50 and 4000 nm in size. They are rich in DNA and histones [119]. Exosomes are vesicles of 20–100 nm size with endosomal origin. The exosomes exist intracellularly within multivesicular bodies which fuse with the cell membrane and then are released into the extracellular space. EVs carry membrane proteins, cytosolic proteins, transcription factors, DNA, mRNA, rRNA, miRNA and various signal transduction molecules. They are rich in heat shock proteins, annexins, cytoskeletal proteins, signal transduction proteins and multivesicular body synthesis proteins. EVs allow intercellular information exchange via different mechanisms such as internalization, ligand-receptor interaction, secreted factors and fusion-mediated transfer of surface receptors, to name a few. Thus, the presence of EVs by themselves in health and disease may be detrimental or beneficial depending on the cell of origin, the cargo they carry and the information they relay [74].

Multiple groups have found a therapeutic advantage of administration of MSC-derived MVs by inhalational or intravascular routes through different mechanisms. Zhu et al. showed that intratracheal instillation of MSC-derived MVs in *Escherichia coli* endotoxin-induced lung injury reduced extracellular lung water, decreased pulmonary edema and lowered lung protein permeability. MVs were also shown to reduce neutrophil influx and macrophage inflammatory protein-2 levels in bronchoalveolar lavage fluid [111]. The same group demonstrated that one of the therapeutic mechanism of MSCs in ALI is mediated through increased production of keratinocyte growth factor (KGF) [109]. Pretreatment of MSCs with KGF siRNA partially eliminated the therapeutic effect of MSC-derived MVs. They concluded that this therapeutic effect is mediated by the transfer of KGF mRNA from MVs to the alveolar epithelium [111].

MVs derived from human MSCs administered to mice injured with bacterial pneumonia decreased the influx of inflammatory cells, cytokines, protein and bacteria. CD44 receptors mediated the uptake of MVs which is essential for the therapeutic effects of MVs [112]. Park et al. tested the therapeutic effects of MVs in an ex vivo perfused human model of bacterial pneumonia. MVs were isolated from the conditioned medium of human BM-MSCs. Administration of MVs significantly increased alveolar fluid clearance and reduced protein permeability and lowered the bacterial load in the injured alveolus. In addition, isolated human alveolar macrophages had increased antimicrobial activity with MV treatment [120].

Chen et al. showed that menstrual blood-derived MSCs exosomes possess therapeutic potential by inhibiting hepatocyte apoptosis in D-galactosamine (D-GalN)/lipopolysaccharide (LPS) induced FHF model in mice, and we further demonstrated that the expression of TNF-α, IL-6, and IL-1β were reduced by co-culture with alpha mouse liver 12 hepatocytes in vitro [121].

In an LPS-induced ALI mouse model, Islam et al. showed that BM-MSCs release mitochondria containing MVs which are then engulfed by injured epithelium improving bioenergetics. The engulfed mitochondrial MVs increased the survival of mice in LPS-induced ALI, and this survival was lost if MSCs contained dysfunctional mitochondria or were depleted of connexin-43 [122]. Similarly, MSCs modulate macrophages in ALI by EV-mediated mitochondrial transfer [123]. MSC-derived MVs contain a substantial quantity of angiopoietin-1 and the immunomodulatory properties of MSCs on macrophages are partly mediated by transferring angiopoietin-1 mRNA to macrophages [124]. Chang et al. demonstrated that intravenous administration of MSC-derived exosomes decreased the levels of TNF-α, nuclear factor-kappa β, matrix metallopeptidase 9 (MMP-9) and IL-1β in the lung parenchyma in a mouse model of sepsis [125].

### Attributes specific to ASCs

ASCs possess qualities that may make them better suited for the treatment of inflammatory lung diseases than other types of MSCs. They display a lower senescence ratio, higher proliferative capacity and are more genetically and morphologically stable in long-term culture when compared with BM-MSCs [126, 127]. In vitro, ASCs are more resistant to apoptosis [128]. Kim et al. demonstrated that ASCs show a significantly greater angiogenic potential when compared with BM-MSCs that suggests they may be effective for the treatment of ischemia associated vascular injuries [129].

Although it was initially shown that ASCs and BM-MSCs exhibit similar immunosuppressive properties in vitro [130], more recent studies suggest that ASCs may be superior to BM-MSCs in this respect as well. ASCs suppress IgG production to a much greater extent [131], and more strongly inhibit the differentiation of monocytes into dendritic cells [132]. When compared with BM-MSCs, ASCs cocultured with peripheral blood mononuclear cells (PBMC) were shown to more strongly inhibit PBMC proliferation and produce lower concentrations of IFN-γ, IL-12 and TNF- α. When cocultured with monocytes, ASC production of IL-10 was more pronounced and resulted in lower levels of IL-6 secretion which led to greater inhibition of immature dendritic cell generation [133]. Collectively, these results suggest that ASCs may be more effective at immune suppression when compared with BM-MSCs.

## Preclinical studies of MSC therapy in Acute Lung Injury (ALI)

MSCs expedite and promote recovery in animal models of ALI which include *Escherichia coli* endotoxin-induced, hypoxia-induced, LPS-induced and ventilator induced lung injury [134–138]. In a systematic review of 17 published preclinical animal model studies that reported on mortality, McIntyre et al. were able to show that treatment with MSCs, as compared to controls, significantly decreased the overall odds of death in animals with acute lung injury [139]. In these models, the mechanism of the repair process is attributed to inhibiting TNF-α release, enhancing IL-10 secretion, decreasing IL-6 levels, increasing KGF, overexpression of angiopoietin-1, reprogramming of macrophage function and secretion of antimicrobial peptides [137, 138].

Systemic and intratracheal administration of MSCs mitigate pulmonary and systemic inflammation as well as enhance bacterial clearance resulting in lower mortality in ARDS [79, 140–148]. As mentioned, MSCs have been shown to have antimicrobial and anti-inflammatory effects [79, 140, 148] and provide benefit to alveolar fluid clearance [109, 149], lung mechanics, and gas exchange [141, 143, 148]. They also minimize distal organ damage [148, 150] and improve survival rate in different ARDS models [79, 80, 139, 140, 143].

Silva et al. directly compared the effects of MSCs with extracellular vesicles (EVs) obtained from those cells involved with lung inflammation and remodeling in a murine model of *E. coli* LPS, induced ARDS. MSCs were more effective than EVs in reducing lung injury. Specifically, MSCs demonstrated superior ability to reduce neutrophil cell count, alveolar collapse, lung elastance, interstitial edema and fibrosis [151]. The safety of intravenous infusion of high dose (10 × 10^6^ cells/kg body weight) BM-MSCs was also demonstrated in an ovine model of bacterial pneumonia [152].

As preclinical evidence of safety and efficacy in larger animals, a canine study of intra-articular injection of allogeneic ASCs was published in 2016 [40]. Although a tissue injection route, this study demonstrated safety in a veterinary clinical setting in a randomized blinded and placebo controlled fashion. Seventy-three dogs were treated with a target dose of 12 × 10^6^ ASCs. Success in the primary outcome variable, Client Specific Outcome Measures, was statistically improved in the treated group compared to the placebo group (79.2% versus 55.4%). The veterinary pain on manipulation score (92.8 versus 50.2) and the veterinary global score (86.9% versus 30.8%) were both statistically improved in treated dogs compared to placebo. There was no detected significant difference between treated and placebo dogs in the incidence of adverse events or negative health findings. An FDA submitted GLP canine preclinical safety study of this same canine ASC product demonstrated safety at a 30X dose level [153]. There were no detectable significant differences in the incidence of adverse events between treated and placebo-controlled dogs.

In a naturally occurring lung disease in horses, ASCs were used to treat lung epithelial and alveolar pathology [154]. Exercise-induced pulmonary hemorrhage (EIPH) occurs in the majority of Thoroughbred and Standardbred racehorses [155–161], as well as other breeds that are required to perform strenuous exercise in their athletic disciplines (e.g. barrel racing Quarter Horses). It can lead to progressively decreased athletic performance [155–161]. EIPH causes persistent structural lung damage with fibrosis and vascular remodeling of the caudodorsal lung fields, as well as venous remodeling and occasional bronchiolar damage [156, 157, 159–162]. This, in turn, leads to pulmonary hypertension, capillary stress failure and hemorrhage into the lung. There is substantial evidence of the ASC’s ability to reduce inflammation, reduce fibrosis and repair damaged tissues [163–166]. Therefore, ASCs were hypothesized to repair damaged vessels, prevent pulmonary capillary hemorrhage, reduce pulmonary hypertension and decrease perivascular inflammation and fibrosis in EIPH. All twelve horses treated with ASCs were able to race again without any prophylactic medications. Prior to treatment with ASCs, all 12 of the horses had obvious epistaxis after racing and veterinary confirmation of lung bleeding by tracheobronchoscopic (TBS) examination, despite having received pre-race prophylactic medications. Following treatment with ASCs, only 1 of 12 horses had epistaxis with exercise. Five of eight horses (62.5%) were confirmed Grade 0 EIPH (no bleeding) by post-race TBS examination. Two horses (25%) were confirmed Grade 1 EIPH, and one horse (12.5%) was confirmed Grade 2 EIPH on post-race TBS examination. Owner/Trainer performance evaluations following treatment with ASCs were reported on only six horses. Four were reported as significantly better, one as better, and one as unchanged. Again, all horses raced without prophylactic medications after treatment with ASCs and no treatment associated adverse events were report.

## The safety of MSC therapy

Intravenous infusion is the most common route for MSC delivery and has been studied in hundreds of clinical trials [167]. Lalu et al. was the first to conduct a systematic review and meta-analysis that comprehensively summarized the safety of intravenous MSC administration in more than 1000 patients [168]. Their analysis was unable to detect an association between MSC treatment and the development of acute infusion related toxicity, organ system complications, infection, malignancy or death [168]. Allogeneic MSC doses up to 100 × 10^6^ have not resulted in serious adverse events with follow-up greater than 1 year in patients with sepsis, ischemic heart disease, perianal fistula, spinocerebellar ataxia, rheumatoid arthritis or severe osteoarthritis [169–176]. Safety and efficacy were also demonstrated in several human clinical trials of immune mediated inflammatory diseases [52–55, 177, 178]. Based upon numerous published human clinical trials, the intravenous administration of BM-MSCs appears to be safe.

The safety of allogeneic ASCs has been studied in cardiovascular disease [179–182], inflammatory bowel disease [183], diabetes mellitus [184–186], kidney disease [187, 188], osteoarthritis [189, 190], bone regeneration [191, 192], cirrhosis [193], multiple sclerosis [52, 194, 195], systemic lupus erythematosus [178, 196–198], graft-versus host disease [177, 199, 200] and acute respiratory distress syndrome [92, 201]. Allogeneic MSC doses up to 100 × 10^6^ have not resulted in serious adverse events with follow up greater than 1 year in patients with sepsis, ischemic heart disease, perianal fistula, rheumatoid arthritis or severe osteoarthritis [169–175].

Toyserkani, et al. conducted a systematic review of adipose derived MSC therapies that included 70 clinical trials and more than 1400 patients with follow-up ranging from less than a month to 3 years [202]. Very few adverse events have been reported that could be related directly to MSC therapy. One case of pulmonary thromboembolism was reported 4 weeks after intramyocardial stromal vascular fraction injection in a patient with New York Heart Association class II heart failure [203]. Henry et al. performed intramyocardial ASC injections in 17 chronic ischemic cardiomyopathy patients with an average ejection fraction of 31% and two patients suffered possible TIA or stroke, but the cause could not be directly attributed to the procedure [204, 205]. These studies did not describe whether the cells were filtered before administration to ensure that the injected cells were single cell suspensions. Thromboembolic complication risk can be assumed to be higher when injecting clumped cells compared with single cell suspensions, particularly when performed in intramyocardial injections. In addition, the patients’ underlying medical conditions must also be taken into account as they had a poor prognosis due to their severe cardiopulmonary disease.

No clinical immune reactions have been seen in studies that have used intravenous ASCs (1 × 10^6^ ASCs/kg bw) [176, 200]. Vaniker et al. [206] demonstrated good safety and improved graft survival when allogeneic ASCs were co-infused with hematopoietic stem cells (HSC) for immunosuppression in renal transplantation patients. However, transient fever has been reported in four randomized clinical trials that used autologous BM-MSCs and one trial of unmatched allogeneic umbilical cord derived MSCs (UC-MSCs) or BM-MSCs [168, 207].

Donor-specific antibodies have been observed in 19–34% of patients receiving allogeneic ASC infusion suggesting that a cellular response can occur. Baseline anti-HLA-I antibodies were seen in these patients possibly as a result of previous pregnancies or transfusions. These patients showed higher frequency of ASC donor-specific antibodies, but no dose-related impact was observed and anti-HLA-II antibodies were not found [171]. The clinical significance of these findings is unknown.

Older adults are at high risk for developing critically severe COVID-19. Tompkins et al. [208] conducted a prospective, placebo controlled study on the safety and efficacy of BM-MSCs for aging frailty in 30 adults with mean age of 75.5 years. Patients were treated with IV infusion of 100 or 200 million allogeneic BM-MSCs. No treatment related serious adverse events occurred at one month follow-up. Better improvements in physical performance, 6-min walk test and forced expiratory volume in 1 s (FEV1) were seen in the 100 million MSC group, but not the 200 million MSC or placebo groups. Immune tolerability was assessed with a panel of reactive antibodies (cPRA) at baseline and 6 months after treatment. One case of mildly increased cPRA was noted in the 100 million MSC group and two cases of moderately increased cPRA were noted in the 200 million MSC group. No clinically significant immune reactions were reported.

The use of cell therapy in patients with active malignancy is of concern, as preclinical in vitro data have suggested that MSC therapy may promote cancer progression [209–211]. However, in the clinical setting this concern has not been realized, possibly because most of the cells do not engraft in the patient. Only one case of cancer recurrence following cell-assisted lipotransfer among 121 breast cancer patients was identified, which is lower than the expected recurrence rate in this population [212]. In a clinical trial of 8 spinal cord injured men, no evidence of tumor development was found after a single IV infusion of 400 million autologous ASCs [213].

Allogeneic hematopoietic stem cell transplantation (allo-HSCT) is often used for the treatment of patients with myelodysplastic syndromes or hematologic malignancies. Chronic graft-versus-host disease (cGVHD) is the leading cause of long-term morbidity and mortality occurring in up to 60% of patients who survive for more than 100 days after allo-HSCT treatment [214, 215]. Gao et al. [61] conducted a randomized, double blind controlled trial on the safety and efficacy of 230 UC-MSC infusions for GVHD prophylaxis after HLA-haploidentical stem cell transplantation in 62 patients with hematologic malignancies. Infusions of 30 × 10^6^ UC-MSCs/100 ml/month) were well tolerated without acute infusion related toxicity or adverse events. The incidence of cGVHD in UC-MSC treated group was significantly lower than the placebo control group (27.4% vs. 48.4%). Survival and disease recurrence did not differ at 51 month follow-up, suggesting that UC-MSC treatment did not increase the incidence of disease recurrence. Interestingly, analysis of a subset of CD4+ CD25+ CD127− regulatory T cells (Treg) and CD27+ memory B-lymphocytes was higher in the UC-MSC treated group although the proportion of total T cells and B cells remained unchanged. Wang et al. [216] conducted a meta-analysis of 6 randomized controlled trials to evaluate the safety of MSC prophylaxis of cGVHD in 365 patients undergoing allo-HSCT. Beneficial prophylactic effects were seen with high dose UC-MSCs but not with low dose BM-MSCs. Prophylactic MSC infusion significantly decreased the incidence of cGVHD without increasing the risk of infection or primary disease relapse. Further, MSC infusion did not impair the anti-tumor and anti-virus T cell responses.

Sepsis is caused by dysregulation of the immune response to infection and is the most frequently observed complication in severe COVID-19 [56, 217]. It is associated with coagulation abnormalities ranging from thrombocytopenia to sustained systemic clotting activation, massive thrombin and fibrin formation and disseminated intravascular coagulation (DIC) with the subsequent consumption of platelets and hemostatic proteins [218]. Significantly greater prothrombin times and D-dimer concentrations were found in deceased COVID-19 patients than those who had recovered suggesting that coagulopathy is associated with poor outcome [219].

Tissue factor (TF), an integral membrane protein, is the most important initiator of coagulation in sepsis [220]. Intravenous administration of MSCs in coagulopathic patients is a safety consideration as ASCs are known to express tissue factor. Compared with BM-MSCs, ASCs demonstrate higher TF expression and reduced hemocompatibility which varies by donor and culture handling conditions [221–224]. For example, cryopreserved ASCs have a stronger effect on coagulation-related gene expression than freshly cultured ASCs [225]. While ASC-associated tissue factor is responsible for systemic activation of the coagulation system, upon intravenous infusion it did not influence microvascular thrombus formation in the lungs in a murine bacterial pneumonia model [225].

Perlee et al. studied the safety and potential use of IV infused allogeneic ASCs in 32 healthy subjects who had received IV purified lipopolysaccharide (LPS), a well characterized model of human inflammation. Patients were injected with up to 4 × 10^6^ ASCs/kg bw. Transient increases in plasma thrombin-antithrombin complexes (TATc) and D-dimer were noted. ASC infusion did not modify plasminogen-activator inhibitor type I (PAI-1) concentrations and inhibited the LPS-induced plasma tissue-type plasminogen activator (tPA) elevations. Clinically, ASC infusions were well tolerated. There were no serious adverse events and arterial oxygen saturations did not differ from the placebo control group. Similar transient procoagulant effects have been demonstrated with bone-marrow derived MSCs and placental derived decidual stromal cells without causing thrombotic events [226].

Liao et al. used heparin therapy to increase the safety of high dose BM-MSC infusion and successfully prevented the coagulation abnormalities caused by tissue factor overexpression in vivo [227]. In humans, anticoagulation protocols have been shown to reduce the risk of thromboembolism in patients with ARDS caused by influenza A H1N1 infection [228]. The clinical relevance of the procoagulant effects of MSCs is unknown, however thrombotic events following administration of MSCs derived from many different tissue sources have not been reported [168]. Concerns regarding coagulopathy with the use of ASCs in ARDS and sepsis patients have not been realized in placebo controlled clinical trials, but these patients typically receive anticoagulation prophylaxis. Matthay et al. [229] studied the safety of IV infusion of allogeneic high dose BM-MSCs in 40 patients with moderate to severe ARDS, 61% of whom had sepsis. They found no MSC related hemodynamic or respiratory adverse events within 6 h of infusion and no evidence for an increased risk of thromboembolic events. However, the potential for MSCs to alter coagulation and induce peripheral microthrombosis, pulmonary embolisms or severe cardiovascular events in high risk patients demands that well-designed studies be conducted to measure the safety of treatments that use MSCs derived from different tissue sources.

## Human clinical trials of MSC therapy in acute respiratory distress syndrome

Zheng recently concluded a phase I, single center, double-blind, placebo-controlled trial assessing the safety of intravenous administration of allogeneic ASCs in patients with ARDS [230]. Twelve patients with moderate ARDS were randomized to receive one IV dose of 1 × 10^6^ ASCs/kg bw or vehicle control. There were no infusion toxicities or serious adverse events and no significant differences in the overall number of adverse events between the two groups. A short-term improvement was observed in oxygenation after ASC infusion, but ventilator-free days, ICU-free days, and length of hospital stay were unchanged.

The results of a phase I, multi-center, open label, dose escalation pilot study (STem cells for ARDS Treatment; START) were reported by Wilson et al. [55]. Nine patients with moderate to severe ARDS received a single intravenous administration of allogeneic BM-MSCs with low dose (1x10^6^ BM-MSCs/kg bw), intermediate dose (5x10^6^ BM-MSCs/kg bw) or high dose BM-MSCs (10x10^6^ BM-MSCs/kg bw). No evidence of infusion-related clinical instability, adverse events or toxicity was observed at any of the doses tested. High dose BM-MSCs improved daily sequential organ failure assessment (SOFA) score compared to lower doses. However, no significant differences in ARDS markers (IL-6, IL-8, ANGPT2, and AGER) were detected in any of the samples collected. Two patients died within 60 days of treatment, but their death was not attributed to the infusion of BM-MSCs.

Simonson et al. tested the IV administration of 2 × 10^6^ cells/kg bw of allogeneic BM-MSCs in two patients with severe refractory ARDS who had failed to improve after all supportive therapies. Both patients recovered from multiple organ failure and presented reduced markers of epithelial apoptosis, alveolar-capillary fluid leakage, pro-inflammatory cytokines, miRNAs, and chemokines in BAL fluid and plasma [229, 231].

Matthay and colleagues reported the results from a prospective, double-blind multi-center, randomized trial (START study) to assess BM-MSC treatment in ARDS [229]. A single intravenous dose of 10 × 10^6^ BM-MSCs/kg bw was compared with placebo in 40 patients with moderate to severe ARDS. Angiopoietin 2 is a well-recognized mediator and biomarker of pulmonary and systemic vascular injury. Angiopoietin-2 concentrations have important predictive value for the development of ARDS [232]. In addition, they robustly predict poor clinical outcomes in adults and children with ARDS and are recognized as prognostic factors in patients with pneumonia [233–235]. There was a strong indirect correlation between cell viability and levels of angiopoietin-2, as well as between viability and improvement in oxygenation index. Angiopoietin 2 levels in plasma showed a significantly greater decrease in the BM-MSC group than in the placebo group [236, 237]. The reductions in angiopoietin-2 concentrations observed in this trial may be related to the release of anti-inflammatory mediators that can mitigate the lung injury. The patients in the BM-MSC treatment group had higher disease severity scores than those in the placebo group at baseline, but mortality did not differ significantly between groups. No patient experienced any BM-MSC-related hemodynamic or respiratory adverse events. The authors concluded that a single dose of intravenous BM-MSCs was safe in patients with moderate to severe ARDS and larger trials were needed to assess efficacy.

In a recent press release [238], Athersys announced positive results of a randomized, placebo-controlled, Phase 2a study which aimed to test the safety and possible efficacy of the adult BM-MSC investigational product MultiStem^®^ in patients with ARDS. Within the prospectively defined group of patients with more severe ARDS, MultiStem^®^ treatment was associated with a markedly greater rate of survival and progression to functional independence at 1 year. As measured at day-28, MultiStem^®^ treatment was associated with a higher mean ventilator-free day (VFD) score of 12.9 vs. 9.2 in the placebo group, and a higher mean intensive care unit (ICU)-free day score of 10.3 vs. 8.1 in the placebo group. As measured at day 28, among more severe ARDS patients, mean VFD in the MultiStem^®^ subgroup was 14.6 vs. 8.0 in placebo subgroup. Mean ICU-free days were 11.4 vs. 5.9 for MultiStem^®^ and placebo recipients, respectively. Quality of Life outcomes, assessed by the EQ-5D, were meaningfully better among all survivors who received MultiStem^®^ treatment compared to those who received placebo. Lower inflammatory cytokine levels at day 7 in the MultiStem^®^ group relative to the placebo group, including IFN-γ, IL-6 and IL-1b among others, suggest the potential for MultiStem^®^ treatment to abate the severe inflammatory response associated with ARDS. MultiStem^®^ treatment was well tolerated in this very sick ARDS patient population, with no serious adverse events related to administration through 1 year of follow-up.

Multiple human clinical studies have reported that MSC administration is safe in patients with severe pulmonary diseases, with little or no infusion reactions and only a few late adverse effects. However, due to the relatively small number of patients that have received MSC therapy thus far, further investigations are required to determine safety and efficacy. The optimal cell origin, cell preparation, cell dose, route of administration and dosing frequency have yet to be determined.

### Human clinical trials of MSC therapy in infectious lung diseases

Recently published evidence by Chen et al., demonstrated promising safety and efficacy data when MSCs were used to treat ARDS associated with Influenza A (H7N9) infection [1]. During the spring of 2013, a novel avian-origin Influenza A (H7N9) virus emerged and spread among humans in China. The disease caused by this virus shares many similarities with COVID-19. Chen and colleagues conducted an open-label clinical trial in 61 patients infected with the H7N9 virus. All patients had moderate to severe ARDS. Seventeen patients consented to receive intravenous infusions of allogeneic menstrual blood MSCs after failure to improve with standard treatment and, in some cases, invasive ventilation and extracorporeal membrane oxygenation (ECMO). Patients were infused with 1x10^6^ MSCs/kg bw, three to four times. No adverse MSC infusion-related events were noted in any of the patients. The clinical outcomes were compared with 44 patients matched with similar symptoms, laboratory tests and other baseline characteristics. There was no significant difference in baseline data between the groups, except that the MSC treatment group had a higher incidence of severe circulatory disturbances prior to treatment. The MSC treated group demonstrated a significantly higher survival rate (82.4%) compared to the control group (45.5%). At discharge, significantly better laboratory test results (procalcitonin, creatinine, creatine kinase, prothrombin time and D-dimer) were noted in the MSC infusion group. At the 1 year followup, chest CT scans of the patients showed significant improvement in the MSC treatment group. At five-year follow-up, all tested survivors from the treatment group did not show any adverse effects of MSC treatment or decline in pulmonary function tests. These promising results support the belief that MSCs reduce the inflammatory effects associated with viral infection, induced cytokine storm and severe respiratory distress. Future placebo-controlled, randomized clinical trials using a greater number of patients are desperately needed to validate these encouraging findings.

### Human clinical trials of MSC therapy in COVID-19

Despite the fact that COVID-19 was first reported only recently, several clinical studies on MSC therapy have been published. Liu and colleagues retrospectively analyzed the differences between 109 COVID-19 patients with and without ARDS. Patients had a mean of age 55 years old with a median follow-up of 15 days. The overall survival rate was 71.6%. Of all the patients, 48.6% developed ARDS. Compared to non-ARDS patients, ARDS patients were older and more likely to have coexistent morbidities. No significant effect on survival was observed in these patients despite the use of antivirus, glucocorticoid, or immunoglobulin treatments [239].

Liang and colleagues [240] reported the treatment of a critically ill 65-year-old female infected with SARS-CoV-2. On January 27, 2020 the patient presented with fatigue, fever and cough. The following day she developed chest tightness, hypoxia and hypertension and tested positive for 2019 novel coronavirus. Radiographs revealed ground glass opacity and 2 days later she was admitted to the hospital. She was initially treated with antiviral therapy (lopinavir/ritonavir), IFN-γ inhalation, oseltamivir, and IV injection of moxifloxacin, Xuebijing, methylprednisolone and immunoglobulin. Her breathing was maintained with a non-invasive mechanical ventilator. Days later she was diagnosed with critically ill type COVID-19 with acute respiratory failure and was transferred to the ICU for ventilator support. Eventually, the glucocorticoid and antiviral therapies were withdrawn, and 1 week later, 50 × 10^6^ allogeneic UC-MSCs were administered intravenously. No obvious adverse effects were noted. The treatment was repeated 3 and 6 days following the initial treatment. Two days after the infusion of her third dose, she was transferred out of the ICU with normal vital signs and laboratory values and a negative throat swab test for COVID-19 antigen. Although this study was limited to just one critically ill patient, the positive outcome supports further investigation.

Leng and colleagues reported similar improvements with intravenous administration of UC-MSCs into seven patients with COVID-19 noting improved functional outcomes and facilitation of recovery [9, 241]. The patients selected were positive for SARS-CoV-2, with one displaying critically severe type, four patients exhibited severe types, and two with milder symptoms of disease. An additional three patients with severe types were enrolled for placebo control. Prior to MSC infusion, all of the patients displayed high fever, shortness of breath, low oxygen saturation and pneumonia. When symptoms worsened, the patients received 1 x10^6^ UC-MSCs/kg bw intravenously and were closely followed for 14 days. Virtually all symptoms subsided within 2-4 days subsequent to MSC infusions with no adverse effects. Chest CT imaging demonstrated that pneumonia infiltration significantly subsided. The majority of patients tested negative for the SARS-CoV-2 nucleic acid test at a week or two after MSC infusion. They found that MSC could significantly improve the functional outcomes of 7 patients without any observed adverse effects. Although no short-term adverse effects were observed, long-term follow up after MSC administration will be necessary in future clinical studies.

The mechanisms underlying the improvement after MSC infusion appears to be the result of robust anti-inflammatory activity. Such processes include an increased number of peripheral lymphocytes, the decline in the C-reactive protein, and decrease of over-activated cytokine-secreting immune cells (CXCR3+ CD4+ T cells, CXCR3+ CD8+ T cells, and CXCR3+ NK cells). Moreover, a group of CD14+ CD11c+ CD11bmid regulatory dendritic cell (DC) population increased after MSC treatment. In comparison to the placebo group, the MSC-infused patients demonstrated a decreased level of TNF-α, and concurrent elevation in the concentration of IL-10, suggesting an improved cytokine milieu. RNA-sequencing showed that infused MSCs were negative for ACE2 and TMPRSS2, which implied that MSCs were free from SARS-CoV-2 infection. Also, the Kyoto Encyclopedia of Genes and Genomes (KEGG) analysis suggested that MSCs were involved in antiviral pathways. The results of these clinical studies in severely ill COVID-19-infected patients show that in vivo administration of MSCs may be a safe and effective approach for treating patients who are in pulmonary distress, including elderly patients with severe ARDS.

Most recently, two commercial companies made press releases of preliminary results of MSC therapy of serious COVID-19 patients. Pluristem announced 100% survival of seven patients with 66% of the patients improving their respiratory parameters [242]. They stated that more studies were planned as well as additional follow-up. No serious adverse events were reported. Mesoblast reported 83% survival in 12 COVID-19 ventilator-dependent patients with ARDS in comparison to 12% survival in patients not given MSC therapy [243]. These preliminary results are encouraging.

## Conclusions

COVID-19 imposes a great public health and socio-economic burden, especially in low-income and middle-income countries. There is an urgent global need for safe and effective treatments. Healthcare services are rapidly becoming overwhelmed by the rise of infected patients who have developed serious medical illness.

It may take months to develop and test a vaccine which, even if effective, will not completely eradicate viral infection. Until other therapeutics become available, cell-based therapies, which have demonstrated safety in human clinical trials, warrant further investigation. We must act now and utilize a scientifically rational approach to care for the rapidly growing number of these patients. First with safety studies, and eventually with larger placebo-controlled, randomized clinical trials. Now is the time for the scientific community to start such studies, so we may offer our patients a viable alternative to standard care.

## Data Availability

Not applicable.
